# Corneal Topography With Upper Eyelid Platinum Chain Implantation Using the Pretarsal Fixation Technique

**Published:** 2015

**Authors:** Ioannis Mavrikakis, Efstathios T. Detorakis, Stefanos Baltatzis, Ioannis Yiotakis, Dimitrios Kandiloros

**Affiliations:** 1 Athens Eye Hospital, Athens, Greece; 2 Department of Ophthalmology, University Hospital of Heraklion, Crete, Greece; 3 1st Ophthalmology Department, University of Athens, Athens, Greece; 4 2nd Otolaryngology Department, University of Athens, Athens, Greece

**Keywords:** Corneal Astigmatism, Corneal Topography, Facial Nerve Palsy, Gold Weight Implants, Platinum Chains

## Abstract

**Purpose:**

To determine the effect of upper eyelid platinum chain implantation, with the pretarsal fixation technique, on corneal astigmatism.

**Methods:**

This is a prospective, cohort study. Fifteen eyes of 15 patients underwent upper eyelid platinum chain implantation, with the pretarsal fixation technique, for facial nerve palsy. Information recorded included patient demographics, etiology for facial palsy, weight of the implant, time from onset of paresis to upper eyelid platinum chain implantation, associated surgical procedures, and preoperative and postoperative corneal topography measurements.

**Results:**

Of the 15 patients studied, 10 were male and five were female. The mean age was 55.9 ± 13.8 years (range, 33–87 years). The most common etiology for facial palsy was acoustic neuroma. The weight of the implant ranged from 0.6 to 1.6gr (median 1.2gr). The time from onset of paresis to upper eyelid platinum chain implantation varied from 1 week to 3 months (median 1 month). Four patients had an associated procedure to correct the effect of paralytic ectropion. There was no statistically significant difference in with the rule astigmatism before and after platinum chain implantation.

**Conclusions:**

Upper eyelid platinum chain implantation, with the pretarsal fixation technique, does not appear to cause significant change in corneal astigmatism. This is contrary to data for pretarsal gold weight implantation, which does induce significant with the rule astigmatism.

## INTRODUCTION

Surgical loading of the upper eyelid helps gravity-assisted closure in paralytic lagophthalmos. Since its introduction by Sheehan (1), several materials have been used, including lead (2), gold (2–8), and platinum (9). Gold is now the most widely used material because of its high density, malleability, minimal tissue reactivity, and color compatibility with skin.

However, implantation of gold weight can affect the contour of the upper eyelid. It could also increase the degree of eyelid-cornea contact. Therefore, it seems plausible that this procedure might alter corneal curvature and affect the patient’s refractive error. A recent study demonstrated that pretarsal gold weights induce a statistically significant increase in with the rule corneal astigmatism (10). In attempts to identify methods of minimizing the induction of with the rule corneal astigmatism associated with surgical loading of the upper eyelid efficiently, we conducted the current study in which we use platinum chains implanted pretarsaly.

The purpose of this study is to evaluate any changes in corneal topography after platinum chain implantation in patients with seventh cranial nerve palsy.

## MATERIALS AND METHODS

This is a single-center, prospective cohort study. All eligible patients undergoing upper eyelid platinum chain implantation for facial nerve palsy at the Athens Eye Hospital, Athens, Greece, were enrolled. Patients were examined over a 3-year period, from 2010 to 2012.

Inclusion criteria were platinum chain implantation, informed consent, and lagophthalmos of 2 mm on forced closure. An exclusion criterion was severe corneal disease preventing reliable corneal topography measurements either preoperatively or postoperatively. As a result, of 20 eligible patients, 18 were enrolled in this study while three were lost to follow-up. We recorded all patient demographics, etiologies for facial nerve palsy, time from onset of paralysis to chain implantation, weight of the chain, associated surgical procedures and preoperative and postoperative corneal topography measurements.

**Figure 1:**
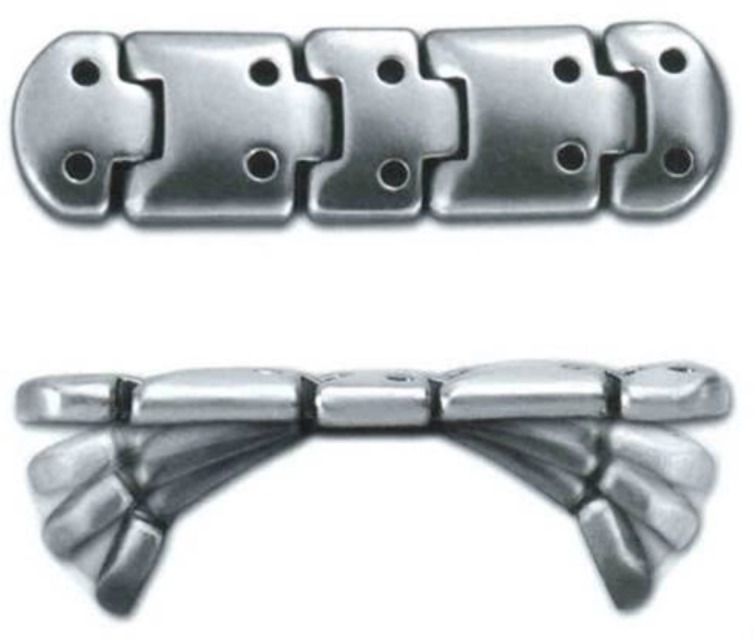
Platinum Chain Implant

All platinum chains (Fig. 1) were implanted by one surgeon (IM). The weight was determined during the preoperative consultation, when it was adhered to the upper eyelid in the area overlying the tarsus with benzoin solution, and eyelid closure was assessed in the upright position (11). The weight was sized up or down as needed to achieve adequate closure without significant ptosis (10).

Surgical procedures were performed under local anesthesia. The approach was through a supratarsal skin crease incision and dissection was carried through the orbicularis muscle to the tarsal plate. A pocket approximately the size of the implant was created over the tarsus, staying at least 2 mm superior to the eyelid margin. The appropriate implant was placed in the pocket, centered medial to the midpupillary line, and sutured directly to the tarsus with two 6–0 Vicryl sutures through the medial and lateral holes. The orbicularis muscle and skin were then closed in layers with interrupted 6–0 Vicryl sutures. Antibiotic ointment was placed over the wound, and a double eye pad applied for 24 hours (10).

Corneal topography was performed with an Eyemap EH-290 (Alcon Surgical Inc., Ft Worth, TX). An absolute scale using 45 colors was used to display dioptric power in 0.25 diopter steps (midpoint value 45.00). We assessed the keratometry (K) readings at 0º, 90º, 180º, and 270º at 3, 5 and 7 mm diameter zones before and after implantation of the platinum chain in the upper eyelid. The amount of with the rule astigmatism was calculated by subtracting the mean of the K readings at 0˚ and 180˚ from the mean of the 90˚ and 270˚ meridians at the 3mm optical zone which is the most visually relevant (10). The change in a vertical steepness was calculated by subtracting the with the rule astigmatism pre platinum chain implantation from the amount post-implantation (10). Corneal topography was performed at least 3 months post insertion of the platinum chain. Most patients required installation of topical lubricants to achieve, where possible, a stable tear film for performing corneal topography. Refractions were not carried out in this study, as it was thought that this particular group of patients with poor blink reflex disrupted tear film, and corneal exposure problems are difficult to assess accurately.

Data were analyzed using SPSS software, version 13 (SPSS Inc.). The significance of the changes in corneal astigmatism was evaluated using a paired T-test while the difference in K measurements for all zones between pre and post platinum chain implantation was assessed using the Wilcoxon Signed Ranks test. Institutional Review Board approval was obtained for this study.

## RESULTS

Of the 15 patients, 10 were male (66.6%), and five were female (33.3%). The mean patient age was 55.9 ± 13.8 years (range, 33–87 years). Tumors caused facial nerve paralysis in 11 cases (10 with acoustic neuroma and one with parotid tumor). There was also one case each caused by cerebral vascular accident, idiopathic Bell’s palsy, Ramsay Hunt syndrome, and polio.

The weight ranged from 0.6 to 1.6 gr (median 1.2 g). The time from onset of paralysis to upper eyelid platinum chain implantation varied from 1 week to 3 months (median 1 month). Four patients had an associated procedure during the upper eyelid platinum chain implantation to correct the effect of paralytic ectropion. Of these, 3 had a lateral tarsal strip and mid face lift, and one had a lateral tarsal strip alone. There was no statistically significant difference in K measurements for any zone between pre and post platinum chain implantation and in the changes in corneal astigmatism. The pre and post-implantation keratometry readings and change in with the rule astigmatism for the 3 mm zone for each patient are shown in Table 1.

## DISCUSSION

In this study, we evaluate any changes in corneal topography after upper eyelid platinum chain implantation in patients with seventh cranial nerve palsy, using the pretarsal fixation technique. Masses arising in the eyelid can cause pressure on the corneal surface with distortion of its topography. Such lesions cause flattening of the globe directly under the area of compression with steepening of curvature adjacent to the area of compression. Eyelid lesions such as hemangiomas and large chalazia may induce central or paracentral compression, whereas more peripheral flattening is induced by masses at or behind the limbus such as dermoids or explants from retinal surgery. The association between astigmatism and upper eyelid abnormalities, such as congenital ptosis (12), hemangiomas (13, 14), and chalazia (15) has been documented.

Pretarsal gold weight implantation also induces corneal astigmatism. More specifically, it was found that, with the rule corneal astigmatism increased significantly by 1.4 diopters ± 2.0, from a mean of 0.3 to 1.7 D after gold weight implantation (p=0.034) (10). The astigmatism is usually mild and can be corrected with spectacles. However, it has been previously found that patient satisfaction following gold weight implantation correlates closely with post-implantation visual acuity (16). Therefore, a surgical technique that reduces induced astigmatism may significantly improve patient satisfaction.

**Table 1:** With the Rule Astigmatism Pre and Post Platinum chain Implantation

Patient	Preoperative Keratometry (Diopters)				With the Rule Astigmatism (diopters)	Postoperative Keratometry (Diopters)				With the Rule Astigmatism (Diopters)	Change in With the Rule Astigmatism (Diopters)
	Axis 0^ο^	Axis 90^ο^	Axis 180^ο^	Axis 270^ο^		Axis 0^ο^	Axis 90^ο^	Axis 180^ο^	Axis 270^ο^		
1	41.96	41.87	37.51	42.86	2.63	41.75	42.24	41.87	42.45	0.535	**-2.095**
2	40.29	46.17	41.15	44.87	4.8	39.99	42.81	39.48	41.07	2.205	**-2.595**
3	42.47	44.18	42.35	43.95	1.655	42.68	43.70	42.30	46.71	2.76	**1.105**
4	45.22	45.89	44.30	47.56	1.965	45.18	48.39	44.15	45.97	2.515	**0.55**
5	43.11	42.63	42.79	42.90	-0.185	41.49	40.20	42.06	42.04	-0.655	**-0.47**
6	42.06	46.12	45.19	47.52	3.195	45.48	48.88	46.31	45.92	1.505	**-1.69**
7	43.78	43.91	43.98	44.37	0.26	43.77	43.99	43.50	46.29	1.505	**1.245**
8	46.05	46.20	47.41	49.05	0.895	48.27	45.23	47.26	48.66	-0.82	**-1.715**
9	46.57	42.15	39.16	42.25	-0.665	41.64	44.77	44.42	47.55	3.13	**3.795**
10	41.57	43.28	41.32	41.64	1.015	44.46	47.42	42.42	40.10	0.32	**-0.695**
11	43.33	39.87	30.58	42.30	4.13	42.55	43.13	38.66	43.99	2.955	**-1.175**
12	47.33	41.14	49.00	43.45	-5.87	40.67	38.78	39.01	38.67	-1.115	**4.755**
13	46.17	47.15	40.12	46.19	3.525	46.10	50.50	45.04	44.63	1.995	**-1.53**
14	39.48	40.41	41.01	46.65	3.285	38.63	40.68	39.51	42.20	2.37	**-0.915**
15	39.55	41.07	38.94	39.65	1.115	39.88	40.04	39.17	40.35	0.67	**-0.445**
Mean					1.45					1.325	**-0.125 ± 2,1**
Pvalue											**p=0.822**

In our study, we found that when using platinum chain implantation with the pretarsal fixation technique, no significant change occurred in the amount of corneal astigmatism (p=0.822). This may be because the platinum chain is flexible and adapts better to the changing radius of the tarsus with movement of the globe, in contrast to gold weights which are rigid (9). Another possible explanation is that the density of platinum differs from that of gold. As a result, for the same weight, the volume of a platinum implant is smaller than that of one in gold. There have been studies reporting the reduction of the side effect of corneal astigmatism using platinum chains with the pretarsal fixation technique (9,17). However, to our knowledge, there have been no studies published describing the effect of upper eyelid platinum chain implantation on corneal topography.

Other methods reported in the literature that have been shown to reduce the side effect of corneal astigmatism use gold weights with the combined high pretarsal and levator fixation technique (18,19). This may be because the position of the implant is above the cornea and not in direct contact with the globe when the eye is open, inducing less corneal warpage (18,19). In conclusion, implantation of a platinum chain in the upper eyelid, in patients with facial nerve palsy, using the pretarsal fixation technique appears to significantly reduce the adverse effect of corneal astigmatism associated with pretarsal gold weight implantation.
